# Characterization of a Novel Association between Two Trypanosome-Specific Proteins and 5S rRNA

**DOI:** 10.1371/journal.pone.0030029

**Published:** 2012-01-12

**Authors:** Martin Ciganda, Noreen Williams

**Affiliations:** Department of Microbiology and Immunology & Witebsky Center for Microbial Pathogenesis and Immunology, University at Buffalo, Buffalo, New York, United States of America; University of Houston, United States of America

## Abstract

P34 and P37 are two previously identified RNA binding proteins in the flagellate protozoan *Trypanosoma brucei*. RNA interference studies have determined that the proteins are essential and are involved in ribosome biogenesis. Here, we show that these proteins interact *in vitro* with the 5S rRNA with nearly identical binding characteristics in the absence of other cellular factors. The *T. brucei* 5S rRNA has a complex secondary structure and presents four accessible loops (A to D) for interactions with RNA-binding proteins. In other eukaryotes, loop C is bound by the L5 ribosomal protein and loop A mainly by TFIIIA. The binding of P34 and P37 to *T. brucei* 5S rRNA involves the LoopA region of the RNA, but these proteins also protect the L5 binding site located on LoopC.

## Introduction

Trypanosomes are unicellular organisms that diverged early in the history of eukaryotic life [1]. Adaptations to parasitism are widespread in this group. African trypanosomes cause disease in mammalian hosts, including humans (trypanosomiasis) and domestic animals (nagana). They present a serious human health issue [2] and are a major cause of morbidity and mortality in the developing world. Trypanosomatids also display several unique cellular and molecular features that make them differ considerably from most well-studied eukaryotes [3].

The assembly of ribosomes is a highly conserved and coordinated process that involves over a hundred accessory proteins [4]. A 45S ribosomal RNA precursor is synthesized in the nucleolus and processed into 5.8S, 18S and 28S rRNAs. 5S rRNA is a small RNA of 120 nucleotides (reviewed in [5]) independently transcribed in the nucleoplasm by RNA polymerase III, transiently bound by the La protein for 3′ maturation and transported to the nucleolus in association with the L5 ribosomal protein [6]. The L5-5S rRNA is the only known eukaryotic extra-ribosomal RNP precursor [7]. Upon incorporation into the ribosomal precursor in the nucleolus, cleavage of the precursor and maturation, the ribosomal subunits are exported to the cytoplasm [8].

Other factors have been described that bind 5S rRNA. Under special circumstances, cells transcribe 5S rRNA in excess and accumulate it in the cytoplasm in storage particles for subsequent mobilization to the nucleolus. This event has been studied in detail in *Xenopus laevis* oocytes [9], where 5S rRNA associates with p43, a 43 kDa protein diverged from TFIIIA.

RNA-binding proteins play essential roles in the many aspects of ribosome biogenesis, including RNA stabilization, processing and transport. Our laboratory has identified two novel, abundant and closely-related RNA-binding proteins in *Trypanosoma brucei* [10], [11]. These proteins, termed P34 (NRBD1, Tb11.01.5570) and P37 (NRBD2, Tb11.01.5590) have been shown to associate specifically with 5S rRNA in nuclear extracts [12]. This association is indicative of potential role(s) of P34 and P37 in the stabilization and transport of 5S rRNA, and in the biogenesis of ribosomes, a process that remains poorly characterized in trypanosomatids. Further work from our laboratory has also shown the involvement of P34 and P37 in the nuclear export of the large ribosomal subunit, 60S [13].

Specific silencing of the expression of P34 and P37 through RNA interference has shown that procyclic cells lacking these proteins exhibit defects in ribosome biogenesis and ultimately lose viability [14]. Specifically, the distribution of ribosomal subunits is skewed towards dissociated particles. This phenotype is accompanied by a substantial and specific decrease in the levels of steady state 5S rRNA [14] and the 5S rRNA still present is not part of high molecular weight complexes. All of this taken together strongly suggests a participation of P34 and P37 in the ribosomal biogenesis pathway, specifically in the binding, stabilization, trafficking and/or ribosomal incorporation of 5S rRNA. Here, we address the interactions between P34, P37 and 5S rRNA at the molecular level.

## Materials and Methods

### RNase H assays

Assays involving radiolabeled RNA were performed as previously described [15]. *T. brucei* 5S rDNA (GenBank M14817.1) was amplified using primers 5ST3Fwd (5′ ATTAACCCTCACTAAAGGGTACGACCATACTTGGCC 3′) and 5SRev (5′ AGAGTACAACACCCCGGGT 3′) to generate a template that was used for T3 polymerase directed *in vitro* transcription (Maxiscript, Applied Biosciences) in the presence of [α-^32^P]UTP.

Full length, radiolabeled 5S rRNA at a concentration of 1 nM was incubated for 20 minutes at room temperature with complementary individual deoxyoligonucleotides targeted against specific secondary structure domains of 5S rRNA (Table 1) and added to the labeled RNA at a final concentration of 1000 nM in 10 mM Tris-HCl pH 7.4, 150 mM KCl, 0.1 mM DTT, 0.1 mM EDTA, 0.1% NP-40. The oligonucleotides used in this assay spanned the 119 nucleotides of the 5S rRNA structure in 12 nucleotide windows (Table 1). RNase H (Applied Biosystems) was added to the reactions (2 U) and allowed to cleave RNA∶DNA hybrids for 15 minutes at 37°C. After addition of 50 mM EDTA, and loading buffer, the enzyme was inactivated by heat and the reactions were loaded onto a denaturing urea 10% polyacrylamide gel. Electrophoresis was performed at 300 V in TBE. Gels were exposed to film with an intensifying screen or alternatively scanned in a phosphorimager apparatus (BioRad). The assay was performed in triplicate.

**Table 1:** Oligonucleotides used in RNase H assays.

Oligonucleotide	Sequence
1	5′ TGGTCGTACCC 3′
2	5′ TTCGGCCAAGTA 3′
3	5′ GGATATGGTGCA 3′
4	5′ ACAAATCGGACG 3′
5	5′ CCGCTTAACTTC 3′
6	5′ CGAGGCCTGTGG 3′
7	5′ TCGCCGTACTAA 3′
8	5′ CGCCATCACTGA 3′
9	5′ CCCGGGTTCCAG 3′
10	5′ AGAGTACAACAC 3′

### Recombinant proteins

Both P34 (GenBank AF020695) and P37 (GenBank AF020696) were cloned into plasmid pQE-1 (QIAGEN) and expressed as histidine-fusion proteins in *E. coli* strain M15. Expression was induced with 1 mM IPTG for 5 hours at 37°C. Cell pellets were frozen and thawed, resuspended in lysis buffer (50 mM NaH_2_PO_4_, pH 8.0, 300 mM NaCl, 1% Triton X-100, 0.5 mM DTT, 10 mM imidazole) and incubated with lysozyme (1 mg/mL) for 30 minutes. Cells were lysed by sonication (six 10 second bursts at 75 W with a 10 second cooling period between bursts) and DNase I was added to a final concentration of 5 µg/mL if the lysate was viscous. After centrifugation at 10,000×g to remove cellular debris, the cell lysate was incubated with a 50% Ni-NTA agarose slurry (QIAGEN) at a 1∶4 slurry∶lysate ratio for 1 hour at 4°C with gentle rotation. The mixture was applied to a column, and washed four times with wash buffer (same as lysis buffer with 20 mM imidazole). The recombinant protein was eluted with elution buffer (containing 250 mM imidazole) in four fractions of 500 µL each. Fractions containing recombinant protein (as visualized by SDS-PAGE with Coomassie blue staining) were pooled and desalted in a PD-10 column (GE) with storage buffer (10 mM Tris, pH 7.6, 150 mM KCl, 0.5 mM EDTA, 0.5 mM MgCl_2_, 1 mM DTT, 0.1 mM PMSF) and flash-frozen in 100 µL aliquots.

### Filter binding assay

A constant concentration of 5S rRNA (equivalent to 10,000 dpm, always lower than 0.5 nM) was used in all the reactions and increasing concentrations of recombinant protein were added in a total volume of 100 µL in binding buffer (10 mM Tris, pH 7.4, 1 mM EDTA, 100 mM NaCl, 0.1% NP40, 100 µg/mL BSA). After incubation for 20 minutes at room temperature, the reactions were loaded onto pre-wetted nitrocellulose filters. The filters were washed twice with buffer, once with ethanol and then dried. A nylon filter underneath the nitrocellulose filters was used to capture unbound RNA [16]. Radioactivity associated with the filters was measured in a phosphorimager. All reactions were performed in triplicate. The bound fraction for each data point was tabulated as a ratio between the signal on the nitrocellulose filter and the total signal on both filters. The dissociation constant was calculated using Graphpad Prism 5.

### Electrophoretic Mobility Shift Assay

Radiolabeled RNA at a constant concentration (approximately 1 nM) was incubated with different concentrations of recombinant protein in binding buffer (10 mM Tris-HCl, pH 7.4, 150 mM KCl, 0.1 mM DTT, 0.1 mM EDTA, 0.1% NP-40) for 20 minutes at room temperature. After incubation, the reactions were loaded onto a 6% native polyacrylamide gel (0.5× TBE, 5% glycerol) and electrophoresed at 100 V for 1.5 hours. Following electrophoresis, gels were dried and exposed to film and phosphorimager screens for signal quantification in a Bio-Rad GS-700 analyzer. The experiments were performed in triplicate and dissociation constants were calculated using Graphpad Prism 5.

For competition assays, the labeled RNA was preincubated with varying amounts of non-labeled RNA and then incubated with protein at a final concentration of 100 nM. Total yeast RNA (Applied Biosystems) was used as a nonspecific competitor. Ribo-oligonucleotides designed to mimic secondary structure elements were as follows: 5′ GCGGGGUGCCAUACUUACAGGCCUCGC 3′ (LoopA/StemV) and 5′GGCCGAAUGCACCAUAUCCCGUCCGAUUUGUGAAGUUAAGCGGCC 3′ (β arm) (IDT). The oligonucleotides were dissolved in 50 mM Tris, pH 7.4, 300 mM KCl, 10 mM MgCl_2_ and heated at 55°C for 5 minutes and allowed to slowly reach room temperature before use. The binding buffer used and the conditions for incubation with protein in these competition assays were identical to those described above.

### RNase H protection assays

Full length, radiolabeled 5S rRNA at a concentration of 1 nM was incubated in the presence or absence of protein (100 nM) in binding buffer (10 mM Tris-HCl, pH 7.4, 150 mM KCl, 0.1 mM DTT, 0.1 mM EDTA, 0.1% NP-40) for 20 minutes at room temperature. Complementary individual deoxyoligonucleotides targeted against specific secondary structure domains of 5S rRNA (Table 1) were added to the RNA at a final concentration of 1000 nM) and incubated for 20 minutes at room temperature. Deoxyoligonucleotide A contains loop A and stem V; C contains loop C and stem III, and III contains stem III alone. RNase H (Applied Biosystems) was added to the reactions (2 U) and allowed to cleave RNA∶DNA hybrids for 15 minutes at 37°C. After addition of 50 mM EDTA, addition of loading buffer and heat inactivation of the enzyme, the reactions were loaded onto a denaturing urea 10% polyacrylamide gel and electrophoresed at 300 V in TBE. Gels were exposed to film with an intensifying screen or alternatively scanned in a phosphorimager apparatus (BioRad) and the experiment was performed in duplicate.

### Mutagenesis of 5S rRNA

A point mutant in Loop C was constructed by site-directed mutagenesis (GeneTailor, Invitrogen) at one of the positions (U43) that interferes with binding of the *Xenopus* L5 protein [17] and with chemical cleavage within loop C [18]. Plasmid pcR2.1Tb5S, which contains the full length rDNA for 5S rRNA, was methylated and subjected to amplification in the presence of an oligonucleotide designed to introduce a single substitution of U at position 43 in Loop C for A. Endonuclease digestion was used to remove the methylated wild type parent plasmid and the amplified plasmid was transformed into *E. coli*. 5S rRNA (ΔU) was generated by *in vitro* transcription as described above for use in filter binding assays.

In addition, we utilized two synthetic β arm oligonucleotides incorporating two relevant mutations in Loop C: the ΔU mutation previously described, and a mutation at a universally conserved G residue at position 41 to a U residue (ΔG). These oligonucleotides were used in unlabeled form in competition EMSAs.

## Results

### *T. brucei* 5S rRNA has a typical secondary structure with a highly accessible Loop C and a structured Loop E

The three dimensional structure of eukaryotic 5S rRNA provides different domains for protein interaction. The 120 nucleotide molecule is typically folded in 5 stems (numbered I–V,) and 5 loops (named A–E) [19]. The structure has three arms branching from loop A. The α-arm consists of stem I, the β-arm comprises stems II and III, as well as loops B and C, and the γ-arm consists of stems IV and V and loops D and E.

It has been shown that TFIIIA binds eukaryotic 5S rRNA via a high affinity interaction with the loop A region, although binding also protects a larger area that extends towards loop E [20]. Eukaryotic L5, on the other hand, binds 5S rRNA mainly through the loop C region [21]. A TFIIIA homologue has not been identified in the *T. brucei* database and although binding of *T. brucei* L5 to 5S rRNA has been identified [22], it has not been characterized.

**Figure 1:**
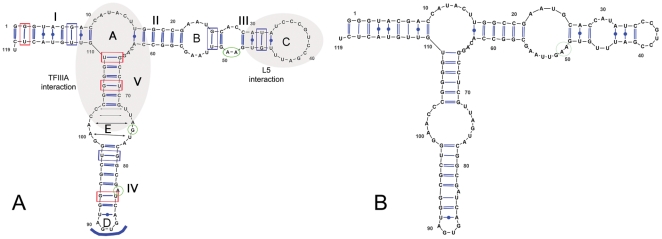
Secondary structure of *T. brucei* 5S rRNA. Mfold was used to generate secondary structures. Boxes indicate G∶U wobble pairs (red: compensated; blue: uncompensated), green circles indicate unpaired purines, blue arc in loop D indicates GNRA tetraloop and arrows in loop E indicate noncanonical interactions between bases in the loop.

In order to examine the interaction of P34 and P37 with *T. brucei* 5S rRNA, we first analyzed the native folding of the RNA *in silico*. Mfold modeling of the *T. brucei* 5S yielded two structures (Figure 1) with similar thermodynamic parameters (ΔG_1_ = −43.30 kcal/mol; ΔG_2_ = −43.10 kcal/mol). The structures differ only in the organization of the β arm. The main structure is depicted in Panel A, and it is typical of other 5S rRNA molecules previously described. There are several G∶U wobble pairs (many of which are uncompensated, i.e. with a pyrimidine 5′ of the G and depicted in blue). In addition, Loop D is a canonical tetraloop (a GNRA motif where G pairs with A, indicated with an arc at the terminal end of Stem IV), and Loop E has the typical eukaryotic signature 5′UUAGUA∶GAACC3′. A G residue is external and unpaired in this Loop (green circle) and the other components of the Loop are usually involved in noncanonical pairings (indicated with arrows in the figure). In contrast, Loops B and C differ considerably between the predicted structures (cf. Figure 1, Panels A and B). A characteristic doublet of unpaired A residues in Stem III flanked by C∶G pairs is present in the first structure but absent in the second one. This pair is postulated to form an A platform [23], a roughly coplanar organization of the purines in which N6 interacts with N3 of the other base via hydrogen bonds [24]. In the second structure, these two A residues form part of an extended Loop B, while Loop C is substantially reduced in size (twelve nucleotides in Loop C of structure A vs. only six in structure B).

**Figure 2:**
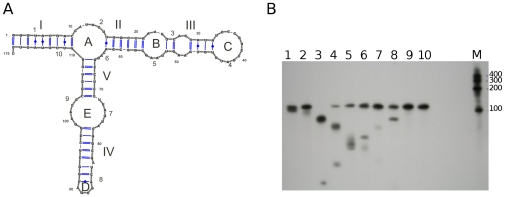
Analysis of the solution structure of *T. brucei* 5S rRNA by RNase H digestion. Panel **A**: Oligonucleotides used and their coverage of the *T. brucei* 5S rRNA structure. Panel **B**: Radiolabeled 5S rRNA was incubated with each of these oligonucleotides and then treated with RNase H. The reactions were then resolved by electrophoresis on a denaturing urea gel.

We first analyzed the solution structure of the *T. brucei* 5S rRNA by RNase H digestion pattern in the presence of specific DNA oligonucleotides (Figure 2). Panel A shows the distribution of oligonucleotides used in the experiment, and Table 1 lists them in the same order. We reasoned that if structure B is prevalent, its shortened stem III and enlarged Loop B would allow a stem III complementary oligonucleotide to pair in this region forming a DNA∶RNA hybrid, which could in turn be cleaved by RNase H. In addition, the shortened Loop C of structure B would make it more difficult for an oligonucleotide directed against Loop C to hybridize and provide a substrate for RNase H. Our results (Figure 2, Panel B) show that loops A, B, C and D are accessible for intermolecular interactions, as demonstrated by RNase H cleavage (lanes 1–6 and lane 8). Loop E (lanes 7 and 10) is not easily accessible for DNA pairing, as visualized by the minimal cleavage obtained with the oligonucleotides that span this loop. This finding is in agreement with previously published data concerning loop E [25] where this motif has been described as a highly structured loop with several intra loop pairings. Critically for discrimination between the two predicted structures, Loop C is the most readily accessible region of the molecule (lanes 3 and 4) as demonstrated by complete cleavage by RNase H after incubation with the Loop C oligonucleotide. In the critical β arm, we also find that incubation with a stem III oligonucleotide does not lead to RNase H cleavage (see protection assays).

### P34 and P37 bind 5S rRNA with high affinity and specificity

**Figure 3:**
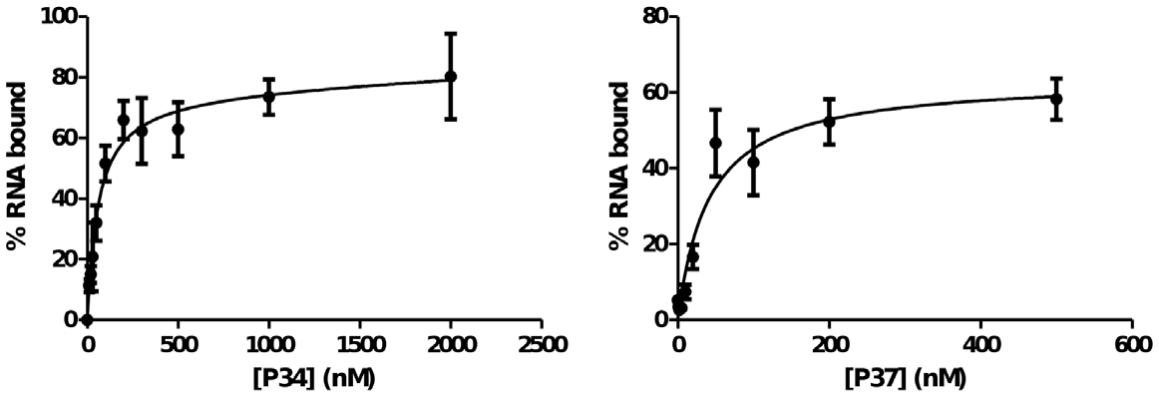
P34 and P37 bind 5S rRNA *in vitro* with high affinity. Recombinant proteins were incubated with radiolabeled 5S rRNA and protein-RNA complexes were analyzed by filter binding assay. The fraction bound to the nitrocellulose membrane is plotted against protein concentration for P34 and P37. The line represents the fit to a bimolecular equilibrium.

It had previously been shown that the trypanosome-specific RNA binding proteins, P34 and P37, associate with 5S rRNA in *T. brucei* [12]. However, it was not clear whether this association represented direct binding of the RNA by P34 and P37, or rather an indirect association mediated by other cellular factors. In order to address this question, we expressed recombinant P34 and P37 in *E. coli* and assessed their binding properties in *in vitro* filter binding assays with radiolabeled 5S rRNA directly (Figure 3). Both P34 and P37 recombinant proteins were capable of binding 5S rRNA in the absence of other cellular factors. Their mode of binding corresponds to that of a bimolecular equilibrium with saturation. The *K_d_* values calculated for the binding reactions are 48 nM for P34 and 40 nM for P37.

**Figure 4:**
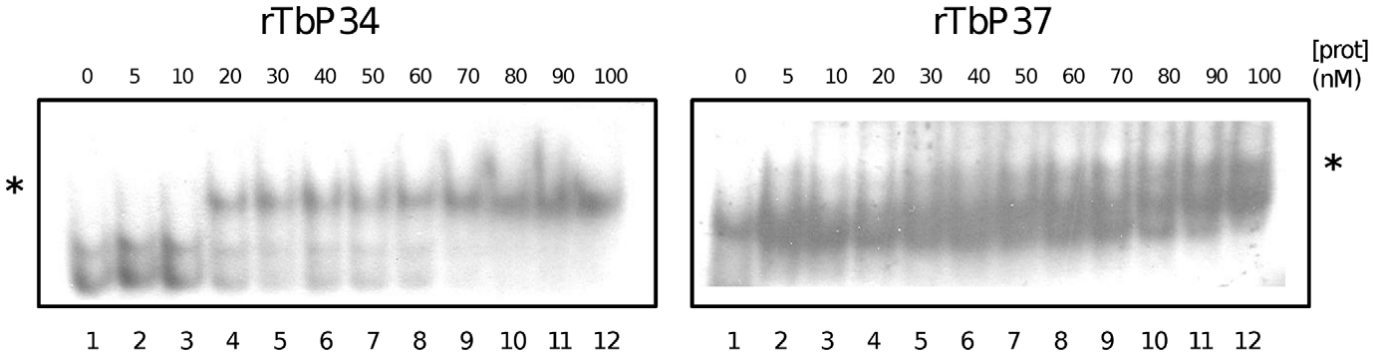
P34 and P37 bind 5S rRNA as a single bimolecular complex. Recombinant proteins were incubated with radiolabeled 5S rRNA and protein-RNA complexes were analyzed by EMSA. The binding reactions were resolved by native electrophoresis. An asterisk indicates the shift.

In addition, we investigated whether P34 and P37 bind 5S rRNA as monomers or multimers since it has been reported that the human 5S rRNA-binding protein L5 can form homodimers [26]. In order to address this question, we performed EMSA with both proteins. Our results show that both P34 and P37 form a single, dose-dependent ribonucleoprotein complex with the target RNA (Figure 4). The first lanes in each EMSA (lane 1, left panel and lane 1, right panel) contain radiolabeled 5S rRNA alone. Subsequent lanes (2 to 12, left and right panels) contain increasing concentrations of protein between 5 and 100 nM. Only a single shift is observed for both proteins as the concentration of protein increases.

**Figure 5:**
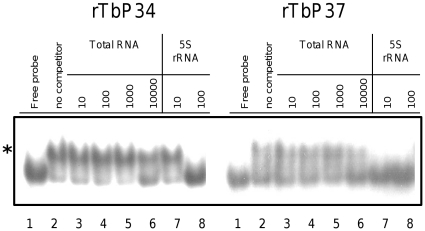
P34 and P37 interact *in vitro* with 5S rRNA with high specificity. Recombinant P34 or P37 were incubated with radiolabeled 5S rRNA in the presence of unlabeled competitor: total RNA as a non-specific competitor (10, 100, 1000 and 10000 ng) or 5S rRNA (10 and 100 ng) as a specific competitor. The reactions were resolved by native gel electrophoresis. An asterisk indicates the shift.

Next, we examined the specificity of the associations by adding the recombinant proteins to a reaction containing radiolabeled 5S rRNA and increasing concentrations of an unlabeled competitor RNA. Total yeast RNA was used as the nonspecific competitor. As shown in Figure 5, the shift in the signal (indicated by an asterisk) observed by the addition of recombinant protein to the radiolabeled probe (lane 2 vs lane 1) is not efficiently competed away by the addition of total yeast RNA when added at 10, 100 or 1000 ng per reaction (lanes 3–5). These amounts represent approximately 30, 300 and 3000 fold mass excess relative to the labeled RNA. It is necessary to use as much as 10000 ng of nonspecific competitor (lane 6) per reaction to observe modest competition of the binding. On the other hand, addition of a specific competitor, unlabeled 5S rRNA, competes for binding very efficiently at only 100 ng per reaction (lanes 7 and 8). The binding to 5S rRNA is therefore not only of high affinity, but also of high specificity, as suggested in previous experiments showing that heterogenous RNA (containing 5S rRNA) was a better competitor than RNA homopolymers [10].

### P34 and P37 interact with 5S rRNA through a high affinity contact with loopA/stemV

**Figure 6:**
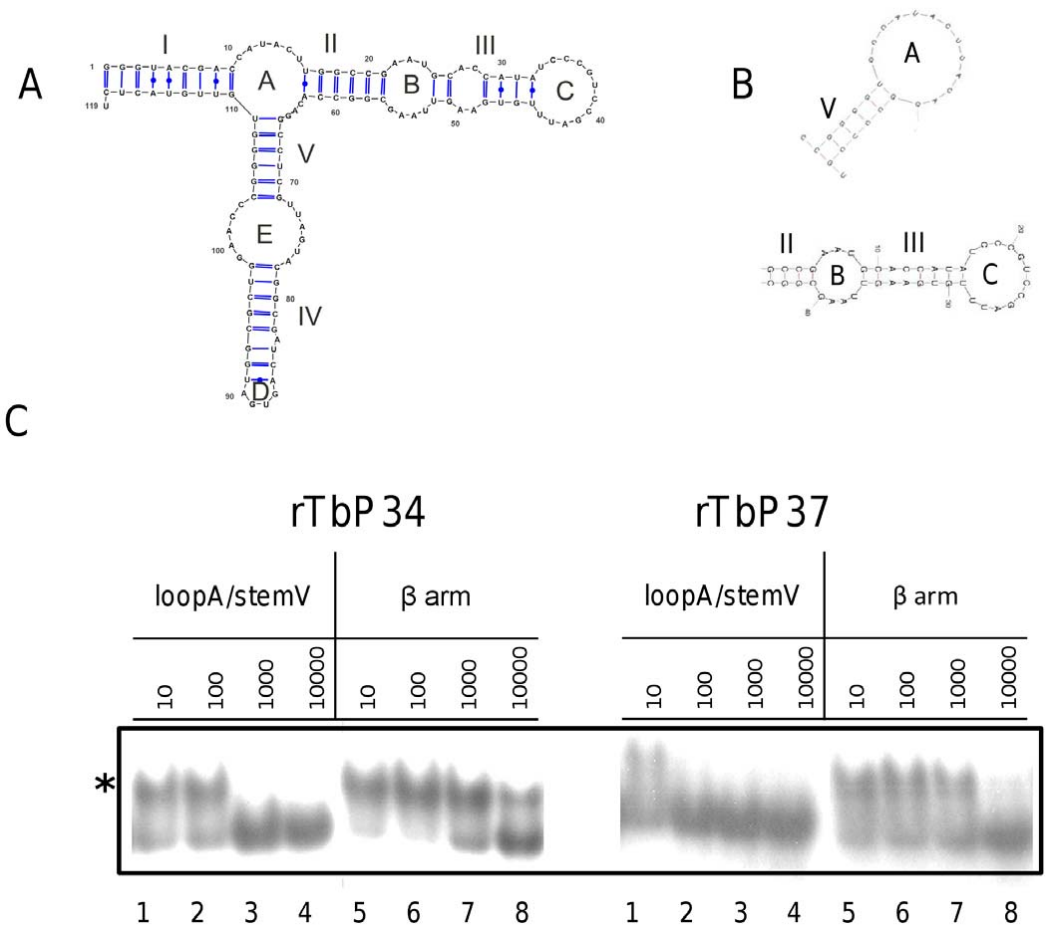
P34 and P37 interact with 5S rRNA preferentially through the LoopA/StemV domain. Radiolabeled 5S rRNA was incubated with recombinant P34 or P37 in the presence of different amounts of unlabeled oligonucleotide competitors: LoopA/StemV and β arm (10, 100, 1000 and 10000 ng). An asterisk indicates the shift.

We wanted to determine the domain or domains on the 5S rRNA interacting with P34 and P37. To achieve this aim, we employed non-labeled oligonucleotide competitors in our EMSAs. The structures of these competitors, as predicted by mfold, are shown in Figure 6, Panel B. The first competitor comprises Loop A and Stem V (containing most of the previously characterized TFIIIA binding region [20]) and the second competitor is a larger molecule(referred to as the β arm) containing Loop C, Stem III, Loop B and Stem II (a domain that includes the previously characterized L5-binding region [27]).

As shown in Figure 6, Panel C, binding of 5S rRNA by P34 or P37 (indicated by an asterisk) was competed away using between 100 and 1000 ng of the Loop A/Stem V competitor (lanes 1–4). However, an additional order of magnitude in the concentration of the β arm oligonucleotide was required to achieve the same extent of competition (lanes 5–8). We obtained similar results using a shorter Loop C competitor (data not shown). Our results indicate that P34 and P37 establish a high-affinity, specific interaction with 5S rRNA via the central portion of the RNA. Interestingly, the Loop A oligonucleotide consistently competes binding somewhat more efficiently in the case of P37 than in the case of P34.

**Figure 7:**
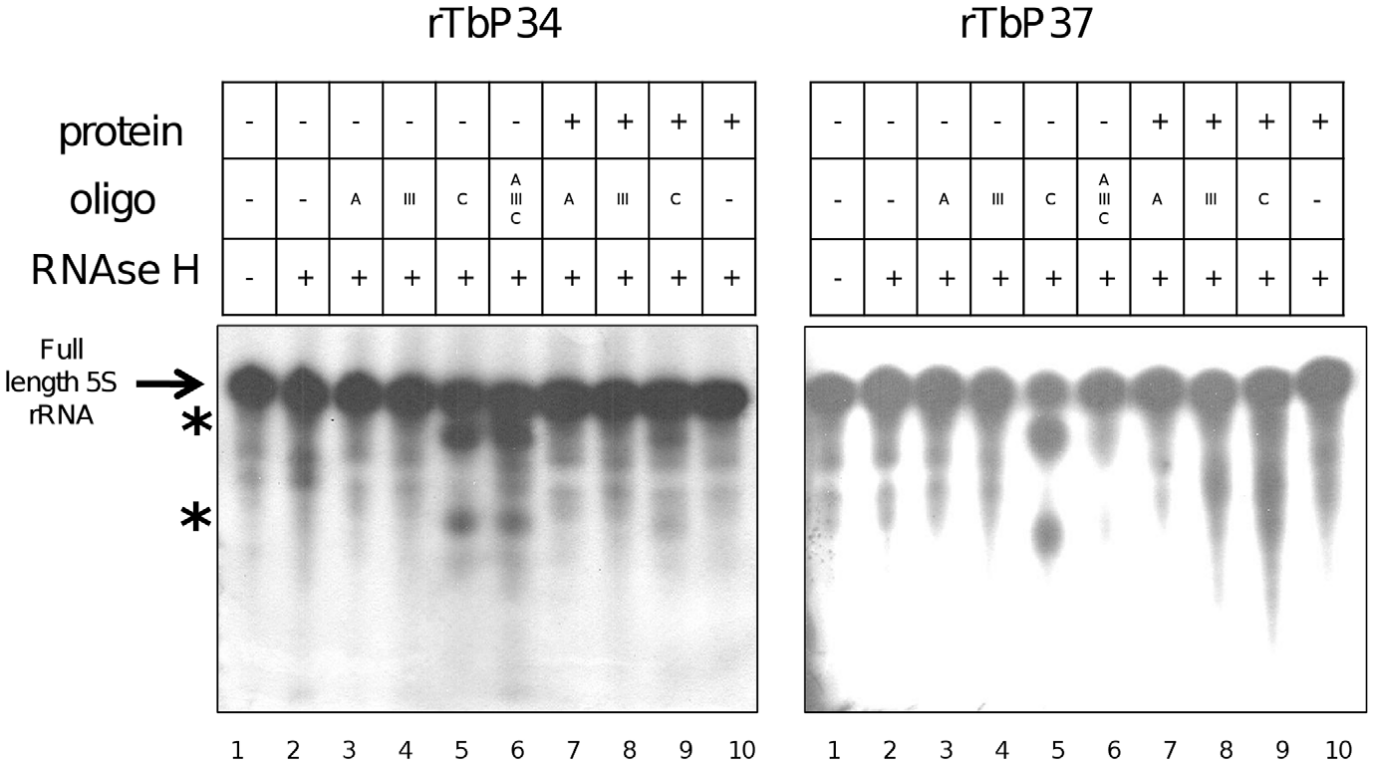
P34 and P37 protect the Loop C of 5S rRNA. RNase H protection assays were performed with full-length 5S rRNA in the absence (lanes 3–6) or presence (lanes 7–9) of protein. The arrow indicates full-length RNA and the asterisks indicate cleavage products.

To further probe the site of interaction on the RNA, we performed RNase H protection assays on full-length 5S rRNA (Figure 7). DNA oligonucleotides designed to hybridize with Loops A or C and with Stem III were annealed to naked RNA (lanes 3–5) or pre-formed RNA-protein complexes (lanes 7–9). Lane 1 contains the control full-length 5S rRNA without RNase treatment or RNA∶DNA hybrids. Lane 2 shows the RNA treated with RNase as a control in the absence of RNA∶DNA hybrids, in which no nuclease-specific cleavage is expected. As a further control, lane 10 contains RNA∶protein complexes treated with RNase, also in the absence of RNA∶DNA hybrids. Finally, the Stem III oligonucleotide is used as a control for a region of the RNA that should not be accessible for annealing in the naked RNA, so cleavage is not expected in the absence or presence of protein (lanes 4 and 8).

When the naked RNA is incubated with the LoopC oligonucleotide and then treated with RNAse, the DNA∶RNA hybrid is formed and RNase H is able to cleave the molecule (lane 5, asterisks). However, if the RNA has been preincubated with P34 (left panel) or P37 (right panel), this region is protected from annealing and cleavage (lane 9 vs lane 5). LoopA, however, is inaccessible to this particular oligonucleotide even in the naked RNA reaction (lanes 3 and 7), presumably because of its short size. Longer oligonucleotides directed against LoopA show that it is available in the naked RNA (Figure 2, lane 6).

Taken together, these results suggest that the P34 and P37 establish the high-affinity interaction with 5S rRNA via the LoopA domain on the RNA, but upon binding protect a region that extends to LoopC. Alternatively, the binding of P34 and P37 could cause a conformation change on the RNA that would make the LoopC region inaccessible to the nuclease.

### Mutating loop C does not affect binding of P34 or P37

**Figure 8:**
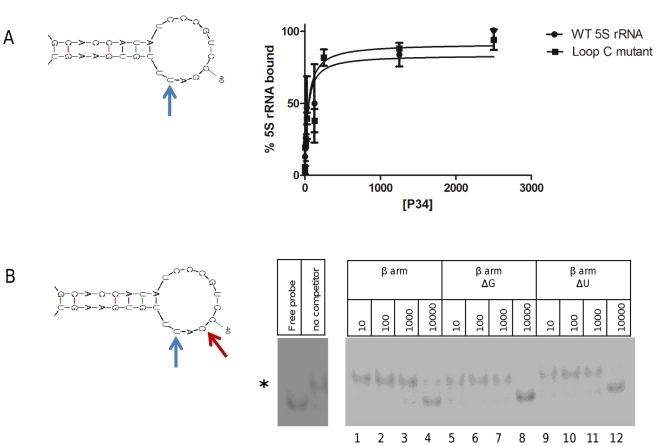
A mutation in T. brucei 5S rRNA loop C does not affect binding to P34 or P37. Panel **A**: A filter binding assay with labeled RNA was performed and binding is expressed in arbitrary units is plotted against protein concentration of P34. Blue diamonds: wild type *T. brucei* 5S rRNA; red squares: loop C mutant. Panel **B**: Competition EMSAs were performed using P34, labeled 5S rRNA and unlabeled competitors: wild type β arm, ΔG mutation (indicated with a red arrow in the structure on the left) and ΔU mutation (blue arrow). An asterisk indicates the shift.

Loop B within the β arm of eukaryotic 5S rRNA does not seem to be involved in the binding of protein factors. The interactions are mediated mainly by Loop C and Stem III. In order to test the functional relevance of loop C in the binding of P34 and P37, we constructed a loop C point mutant in which position 43 has been changed from U to A (indicated with a blue arrow in Figure 8, Panel A), preserving the structure of the loop. This ΔU mutation has been shown to decrease binding of *Xenopus* L5 [17]. In addition, the same mutation was shown in a different study to alter the cleavage pattern within loop C [18]. We used this mutant form of *T. brucei* 5S rRNA and wild type 5S rRNA in filter binding assays with P34 and the results show (Figure 8, Panel A) that no significant differences are observed between the wild type and ΔU 5S rRNA molecules in their binding to P37. Similar results are obtained with P37 (data not shown). This is in agreement with the competition experiments that show that loop C is not directly involved in the interaction with P34 and P37.

The 5S rRNA β arm ΔU mutation was used also in competition EMSAs with wild type labeled 5S rRNA and P34 (Figure 8, Panel B). When compared to the effect of a wild type unlabeled β arm competitor (lanes 1 to 4), no significant differences are observed (lanes 9 to 12). Both the wild type β arm and the ΔU mutant compete for binding of P34 to labeled full length 5S rRNA at 10000 ng per reaction. Furthermore, we analyzed the effect of a mutation in a universally conserved G residue within Loop C (indicated with a red arrow in Figure 8, Panel B). A substitution in this position did not affect the properties of the competitor, as demonstrated in lanes 5 to 8.

## Discussion

The secondary structure of 5S rRNA is largely conserved across all three domains of life [28]. *T. brucei* 5S rRNA does not differ significantly from other eukaryotic 5S rRNA molecules in primary or secondary sequence (Figure 1). In particular, several important domains and signatures can be identified. A three-way junction gives 5S rRNA a characteristic Y shape and it provides a compact arrangement rich in secondary structure motifs. The distinct element Loop E [25] is well conserved and typically eukaryotic in the *T. brucei* 5S rRNA. The Loop D tetraloop with a single preceding bulge [29] is also present and the overall structure contains the typically high proportion of uncompensated G∶U wobble pairs. These tend to destabilize the helix by providing a discontinuity in the stacking of bases, making them ideal as initiators of an internal loop (as with the invariable G∶U wobble pair at the base of the three way junction of Loop A) and potential sites for protein interactions [30]. Interestingly, another site which can potentially interact with protein factors, two consecutive unpaired A residues in Stem III, are present in one of two possible predicted structures for *T. brucei* 5S rRNA. This A platform is in the part of the molecule that constitutes the L5 binding motif in *Xenopus* [27]. Our RNase H assays address the accessibility of different portions of the molecule to the solvent and their ability to interact with nucleic acid. We provide evidence that favors the presence of structure 1 by demonstrating that Loop C is the most readily accessible element of the molecule as shown in Figure 2. Furthermore, stem III is not easily accessible in our RNase H assays (Figure 7, lane 4). However, one must bear in mind that the ability of a single-stranded region of RNA to hybridize with a DNA oligonucleotide may not be constant in all environments. Extensive intraloop hydrogen bonds could prevent typical Watson Crick pairing to a DNA oligonucleotide (and this is, in fact, observed for the Loop E region). In addition, we sometimes observe multiple forms of the RNA in native EMSAs in the absence of protein, indicating the presence of more than one structure, at least under certain conditions. The only structural element commonly found in 5S rRNA molecules which we were not able to identify in our predicted models was a conformationally flexible bulged residue in Stem II [31], [32].

We demonstrate here that two trypanosome-specific proteins, P34 and P37, participate in a direct interaction with 5S rRNA (Figure 3). The dissociation constants for these proteins are 48 nM and 40 nM for P34 and P37, respectively. Each protein forms a single complex with labeled 5S rRNA (Figure 4). These interactions are specific and they are not a result of general RNA binding characteristics of the proteins (Figure 5). This finding expands on previous results from our group indicating a lack of affinity of P34 and P37 for RNA homopolymers and dsDNA, [10] and no *in vivo* association with 18S or 28S rRNAs [12]. Results from competition experiments (Figure 3) show that P34 and P37 bind the RNA through a high affinity interaction with the Loop A/Stem V domain. Therefore, these proteins bind a region different from the Loop C domain, a canonical protein binding site for L5 [21]. However, we have also shown that the footprint of binding is rather large, and the Loop C is protected by the binding of P34 or P37 to 5S rRNA (Figure 7). This may preclude other RNA-protein interactions to occur via this domain. A large protection area in protein∶RNA interactions for 5S rRNA is not uncommon, and there is overlap in the protection areas of TFIIIA and L5 [33].

P37 seems to display more affinity for the Loop A region than P34 (Figure 3, lanes 1–4 left side vs. right side). The main difference between P34 and P37 is an N-terminal stretch of 18 amino acids present in P37 and absent in P37. Since we did not detect significant differences in the affinity of the proteins for full-length 5S rRNA (or in any other *in vitro* assay), the significance of this observation is unclear at the moment. It could be that P34 relies on additional contacts elsewhere on the RNA.

Finally, we sought to further examine the role of Loop C in the binding of P34 and P37. If this region is merely protected by the proteins but is not directly involved in the binding, we would predict that mutations within this Loop would be well tolerated. We selected two mutations: one that has been used in previous work with *Xenopus* L5 and reduces binding of 5S rRNA to the protein, and a mutation in a universally conserved G residue within the loop. The fact that we detected no differences in binding between the mutant and the wild type RNAs (Figure 8) strengthens the hypothesis that binding involves determinants elsewhere in the molecule, namely in Loop A and Stem V.

The intriguing presence of the trypanosome-specific factors in an association with the highly conserved 5S rRNA raises questions concerning the role of these 5S rRNA binding proteins relative to the other well characterized 5S rRNA binding proteins, TFIIIA and L5. No TFIIIA homologue has been described or identified by data mining to date. It is unclear whether P34 and P37 may perform some of the functions of TFIIIA, but given the major structural differences between these proteins (specifically the lack of zinc finger domains universally found in TFIIIA) we consider this possibility unlikely. Furthermore, the protection of Loop C by P34 and P37 does not suggest a TFIIIA-like mode of binding to the rRNA. The *T. brucei* genome contains a gene for the L5 ribosomal protein, and its presence has previously been shown in association with 5S rRNA [22]. A preliminary analysis of the primary sequence of *T. brucei* L5 reveals potentially significant deviations from the eukaryotic consensus, but the significance of this is not yet clear (data not shown). As indicated above, the binding sites of P34 and P37 and L5 are not the same, although P34 and P37 binding does protect part of the L5 binding site.

Previous data from our laboratory using RNA interference demonstrated that *T. brucei* cells lacking P34 and P37 exhibit a marked reduction in the stability of 5S rRNA [34] and that assembly of ribosomes does not proceed normally. This phenotype is reminiscent of what is observed in *Saccharomyces cerevisiae* when L5 is knocked out [35]. The functions of P34 and P37 may therefore partially overlap with those of L5 in *T. brucei*. As previously indicated, L5 has been detected in association with 5S rRNA. If this association also involves P34 and P37, our data would suggest that the addition of L5 to the P34/P37:5S rRNA complex might take place through protein-protein interactions, rather than protein∶RNA interactions (given the large protection footprint of P34 and P37 on 5S rRNA). A trimolecular complex could be formed by a protein∶RNA interaction and a subsequent conformational change to allow for the binding of the second protein partner in the complex, in a manner analogous to the formation of the 42S complex in *Xenopus*. The *Xenopus* storage particle component p43 binds 5S rRNA primarily through interactions in the Loop D region [36] and then associates via protein∶protein interactions with p50, forming the 42S particle. The molecular interactions between P34, P37, 5S rRNA and the L5 protein are being currently dissected in our laboratory.
